# Structural and Electrochemical Properties of 4‑Methyl-4′‑(*n*‑mercaptoalkyl) Biphenyls Self-Assembled on the Au(100)–​(1 × 1) Surface

**DOI:** 10.1021/acs.langmuir.5c02088

**Published:** 2025-07-29

**Authors:** R. Aguilar-Sánchez, Yongchun Fu, M. Homberger, U. Simon

**Affiliations:** † Facultad de Ciencias Químicas, 3972 Benemérita Universidad Autónoma de Puebla, 72570 Puebla, Mexico; ‡ Institute of Inorganic Chemistry, RWTH Aachen University, 52074 Aachen, Germany; § Department of Chemistry and Biochemistry, University of Bern, CH-3012 Berne, Switzerland

## Abstract

Scanning Tunneling Microscopy (STM) and electrochemical studies were conducted on a series of 4-methyl-4′-(*n*-mercaptoalkyl) biphenyls CH_3_(C_6_H_4_)_2_(CH_2_)_*n*_SH (BP*n*, where *n* = 1–6) self-assembled on the unreconstructed Au(100)–(1 × 1) surface. The study investigated the role of substrate crystallography in the electrochemical and surface properties of BP*n* adlayers. Notably, unlike BP*n* on Au(111)–(1 × 1), short alkane chain BP*n* (1 to 4) does not induce gold surface vacancies when adsorbed on the Au(100)–(1 × 1) indicating distinct surface chemistry. The adlayers exhibit two coexisting phases (α and β), the presence of which evolves with the number of methylene units. The electrochemical properties of the BP*n* adlayers are characterized by higher thermodynamic stability during reductive desorption with charge values alternating with the number of methylene units. The molecule adsorption modifies an important parameter such as the work function of the underlying Au(100)–(1 × 1) metal substrate. The reciprocal capacitance exhibits a linear dependence on the length of the alkane spacer. Its change per CH_2_ group agrees well with the values of *n*-alkanethiols on Au(111).

## Graphical abstract

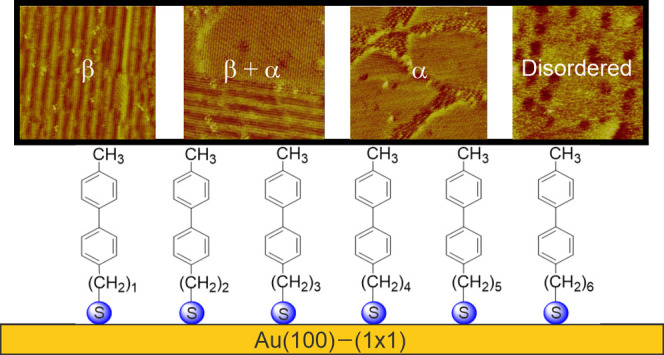

## Introduction

Molecular self-assembly represents a versatile approach to design and functionalize surfaces governed by the subtle balance between intermolecular forces and molecule–surface interactions. Surface properties can be tuned by the appropriate choice of substrate materials and chemical functionalities. The functionalization of solid surfaces by self-assembly processes has become increasingly significant for their application in various fields of current research including molecular electronics,^1,2^ surface science methods,^3^ biomolecular devices,^4^ insulating materials,^5^ biosensors,^6^ nanofabrication and nanolithography,^7^ and interestingly as coatings in batteries.^8,9^

Due to their flexibility in the design of molecular structures and simple preparation methods, thiol-based organic monolayers have been widely recognized as convenient systems for controlling charge transfer at metal surfaces.^10^ The ability of self-assembled monolayers (SAMs) to finely tune properties such as the work function,^11,12^ phase transitions,^13^ and dynamics^14^ at the electrode surface has been widely researched on Au(111) in high contrast to the thiolate–Au(100) interface. Understanding how the substrate influences adsorption, stability, and reactivity in molecular adlayers, it is essential to design surface properties with optimized functionalities through different thiol–Au­(*hkl*) interactions.^15^

Significant efforts have been made to explore alternative approaches to the Au(111) substrates. Alkanethiols on Au(100) including ethanethiol,^16^ propanethiol,^17^ butanethiol,^18^ and decanethiol^19^ have been extensively studied. Extensive electrochemical STM studies have revealed that vacancies—one of the most notable features of alkanethiol-covered Au(111) surfaces—are absent in all studied alkanethiols on Au(100).^16–18^ Furthermore, with the exception of decanethiol,^19^ all tested alkanethiols on Au(100) developed a high density of gold islands following molecular adsorption, with two distinct molecular phases. Additionally, the electrochemical stability of alkanethiols, along with other anchoring groups such as selenols, is higher on Au(100). This enhanced stability is attributed to the electronic distribution surrounding the Au–S bond, which varies depending on the crystalline orientation and the chemical environment of the adsorbates, particularly the hybridization of the carbon atom bonded to sulfur.^20^ The influence of Au(100) coordination sites on adsorption surface energy has also been explored from theoretical studies,^21,22^ providing deeper insights into surface interactions at the molecular level. The Au(100)–(hex) surface is particularly interesting due to its hexagonal reconstruction in the topmost atomic layer, which can be transformed into a square atomic arrangement of gold atoms Au(100)–(1 × 1), through the adsorption of thiols^19^ or anions under potential control.^23^ Inspiring experiments using STM, X-ray photoelectron spectroscopy (XPS), and electrochemical techniques have demonstrated that the structural and electrochemical properties of hexanethiol SAMs can be tuned by lifting the Au(100) surface reconstruction prior to thiol adsorption.^24,25^ While the reconstructed Au(100)–(hex) surface showed rectangular islands nucleated on terraces following hexanethiol adsorption, the Au(100)–(1 × 1) surface displayed only smooth terraces and striped molecular domains. This contrast highlights the significant influence of surface reconstruction on molecular organization and adsorption behavior.

Despite aromatic thiols representing model systems for enhanced conductivity and control of defects due to the rigidity of phenyl rings, they have been far less studied than alkanethiols.^26–28^ Studies performed, for example, with 6-mercaptopurine^29^ adsorbed on Au(100), have shown that molecular disorder is minimized due to reduced degrees of freedom and the presence of more mobile species on this surface compared to Au(111). Previous studies^30–35^ performed on biphenyl–alkanethiols have revealed a pronounced influence of the S–Au interface on the monolayer structure, with notable variations in molecular orientation and electrochemical properties depending on the length of the alkane spacer on Au(111). However, the impact of substrate crystallography on these properties has not been studied.

In this context, a detailed knowledge of the structure and electrochemical properties of surface ligands, such as thiols, on different substrate facets is crucial for the understanding, design, and synthesis of nanostructured surfaces with optimal tailored functionalities. For instance,^36,37^ a gold nanoparticle catalyst consists of approximately 70% (111) facets, while the remaining 30% comprises (100) or (001) facets. Selective desorption of chemical entities from a surface could serve as a simple and reliable method for fabricating functional surfaces designed to be electrocatalysts or sensors, by selectively exposing either the (111) or (100) facets to the electrolyte. These findings are particularly relevant to have a comprehensive knowledge of, for example, thiolated Au nanoparticles where the (100) or (111) faces are the most dominant and catalytically active. The most common approach to protecting metal nanoparticles involves the use of thiolate SAMs, which play a crucial role in stabilizing and functionalizing these nanostructures.

Building on our efforts to understand the molecular factors underlying the electrochemical properties of biphenyl SAMs, here, we present structural and electrochemical studies of a homologous series of biphenyl thiol SAMs on the unreconstructed Au(100)–(1 × 1) surface. These thiols which are characterized by a biphenyl unit linked to a thiol group by an alkane spacer of varying length (see Scheme 1) exhibit a pronounced dependence of their structural and electrochemical properties on the number of methylene units. Additionally, their properties are strongly influenced by the crystallography of the substrate. In this work, we aim to identify the crucial role of the substrate crystallography in shaping the surface chemistry of metal–thiol interactions, as well as its effects on the organization, adsorption, stability, and charge transfer properties of aromatic biphenyl–alkanethiol SAMs using the defect-free Au(100)–(1 × 1) as a substrate. The findings of this study aim to contribute to a deeper understanding of these interactions, which are significant owing to their decisive role in the design of catalysis and materials engineering.

**Scheme 1:**
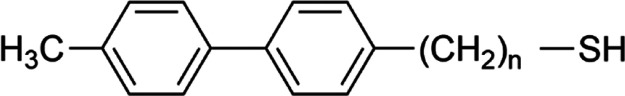
Molecular Structure of BP*n* (Where *n* = 1–6) (4-Methyl-4′-(*n*-mercaptoalkyl)­biphenyl)

## Experimental Section

### Sample Preparation

The substrates used in this work were massive Au(100) single crystals from MaTeck Jülich (Germany). Before depositing the BP*n* molecule, the electrode was annealed in a butane flame at red heat for 10 min and then cooled down under an argon atmosphere to room temperature. Defect-free Au(100)–(1 × 1) surfaces were prepared by immersing the flame-annealed Au(100)–(hex) electrode into deaerated 5 mM HCl (Suprapure, Merck Germany) solution under potential control^30,38^ at 0.5 V (vs SCE). After 10 min, the electrode was removed from the electrochemical cell, rinsed with a copious amount of ultrapure water (18.2 MΩ, 2 ppb TOC), and dried with hot absolute ethanol. Biphenyl-thiols were prepared according to Ref 35. Biphenyl-thiol SAMs were formed by immersing a defect-free Au(100)–(1 × 1) electrode into a 1 × 10^–4^ M BP*n* solution in ethanol. The samples were then annealed in a tightly sealed, oxygen-free container for 12 h at 90 °C under an Ar atmosphere. The precise control of the assembly conditions, including temperature, concentration, and time, is critical for fabricating ordered adlayers of large thiolated molecules.^39^ After assembly, the modified surfaces were rinsed with hot ethanol and transferred to either the electrochemical or STM cell.

### Electrochemical Studies

All electrochemical measurements were performed with an Autolab PGSTAT 30 workstation in a conventional three-electrode glass cell. The BP*n*–Au­(100)–(1 × 1) surfaces were used as working electrodes, and the counter electrode was a platinum wire and a saturated calomel electrode serving as the reference. All electrolyte solutions—NaOH, HClO_4_, KClO_4_—were prepared from Suprapure substances from Merck (Germany) in Milli-Q ultrapure water (18.2 MΩ, 2 ppb TOC). A mixture of K_4_Fe­(CN)_6_ and K_3_Fe­(CN)_6_ (Fluka, puriss. p.a.) at a 1 mM concentration was used as the redox probe in the electron-transfer studies. The electrolytes were purged with Ar for 20 min before and during the experiment. All glassware was cleaned in a hot 1:1 mixture of H_2_SO_4_ (95–97% pro-analysis, Merck) and HNO_3_ (65% puriss. Riedel-de-Haen), followed by copious rinsing with ultrapure water.

### Scanning Tunneling Microscopy Studies

The STM studies were carried out with BP*n* monolayers on Au(100)–(1 × 1) single-crystals (see Sample Preparation) in decane, employing a Nanoscope IIIA SPM (Digital Instruments). The tips were mechanically cut into Pt_0.8_Ir_0.2_ alloy (99.99%) wires. All measurements were performed in constant current mode, using low tunneling currents (50 to 300 pA).

## Results and Discussion

### BP*n* Adsorption on Au(100)–(1 × 1)—STM Studies

The structural properties of freshly prepared BP*n* monolayers were studied by STM. Low-resolution STM images of BP*n* (*n* = 1–4) are presented in Figure 1, following a careful assembly process. The images show features consistent with high monatomic gold islands^40,41^ exhibiting square-like and patched structures on the terraces of Au(100)–(1 × 1), with a higher density for BP1 and BP3 samples. During the lifting of the hexagonal reconstructed phase, 24% of the extra gold atoms from the topmost layer are expelled, forming numerous monatomic-high gold islands on the surface. If the reconstruction is lifted under electrochemical potential control, the islands coalesce decreasing in density, and only a few rounded gold islands remain on the surface.^42^ These features remain visible after BP*n*–thiol adsorption, but their shape changed from rounded to square type and their amount increases, particularly for BP1 and BP3. Since the Au(100)–(hex) surface reconstruction was lifted before the BP*n* adsorption, the gold adatoms nucleate and grow on the terraces. However, the increased number of islands suggests that not all of them originated from lifting of the surface reconstruction. Instead, they could be considered as a result of an adsorbate-induced reconstruction,^16,17^ which is more prominent for BP1 and BP3.

**Figure 1:**
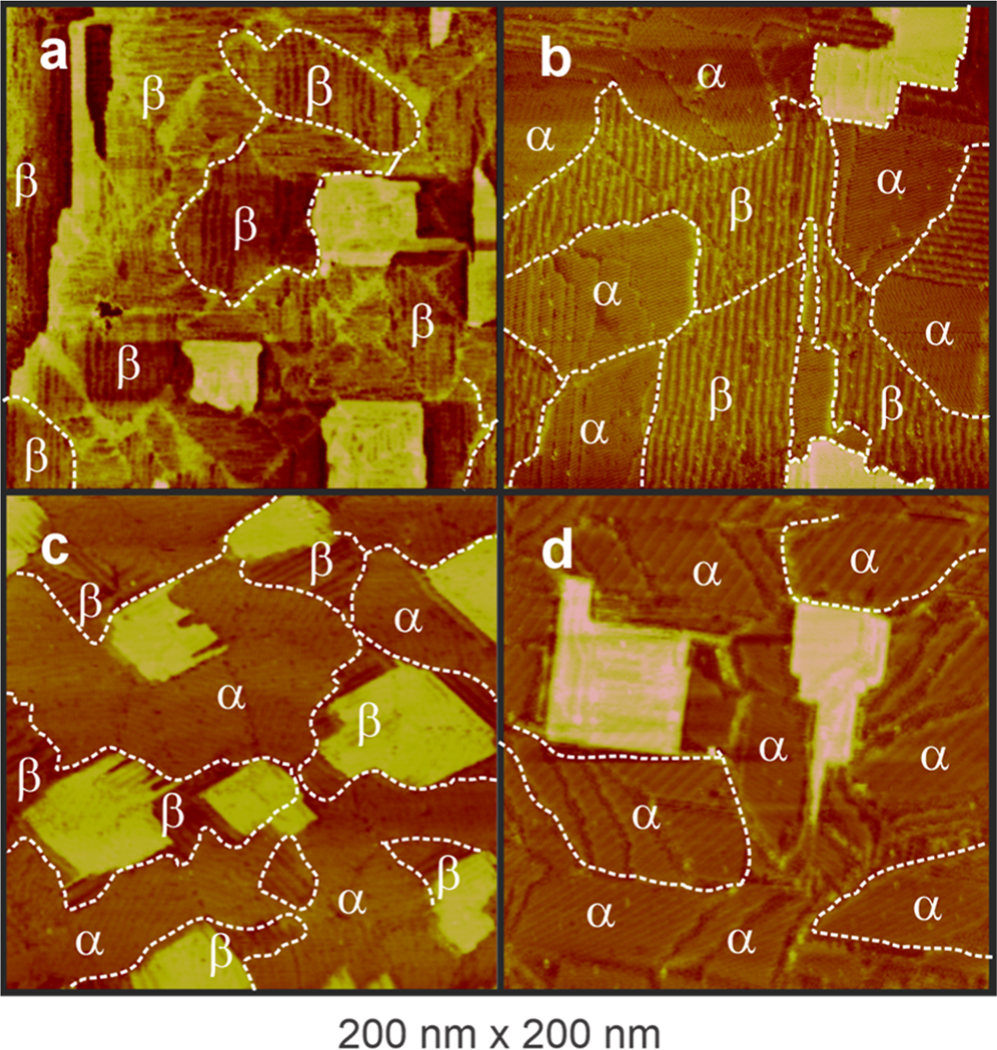
Low-resolution 200 nm × 200 nm STM images of BP*n* (*n* = 1 to 4) on the Au(100)–(1 × 1) surface. BP1 (a) and BP3 (c) show terraces with high density of the square-type gold island on the surface. BP2 (b) and BP3 (c) show the formation of two phases; α and β. BP1 (a) displays domains exclusively of the β-phase, whereas BP4 (d) shows the formation of only the α-phase. BP2 (b) and BP4 (d) display lower density of gold islands. STM parameters: bias voltage 200 mV, tunneling current 0.1 nA.

A closer inspection of the monolayers (Figure 2) reveals interesting characteristics. Monolayers of BP1 to BP3 exhibit two distinct structures: a striped phase (β) was predominantly observed in BP1 and BP3 (Figure 2a,c), while a second quadratic phase (α) was also found in a few domains. In BP2, the α phase was predominant and it was the only phase observed in BP4 (Figure 2b,d). Molecular resolution STM studies (Figure 3) of the α phase, conducted for BP2 and BP4 (Figure 3a), revealed a semiquadratic (2 × 3) structure with long-range ordering of individual molecules (Figure 3a,c), consistent with a compact molecular arrangement on the Au(100)–(1 × 1) surface. In contrast, the β phase exhibits a less-ordered structure (Figure 3b,d), making it challenging to propose a definite structural model. However, the unit cell parameters (0.6 nm × 4.1 nm) suggest a tentative structure of (10√2 × 1.5√2) which is incommensurate with the substrate and likely consists of molecules more tilted with respect to the substrate plane compared to those in the α phase. Since STM cannot directly reveal the structure of the metal Au(100) surface in the presence of the BP*n* SAM, further studies using other surface characterization techniques are necessary to confirm this hypothesis. XPS studies could allow determination of the chemical state of sulfur, variations in molecular orientation with chain length, and different binding geometries or interactions.

**Figure 2:**
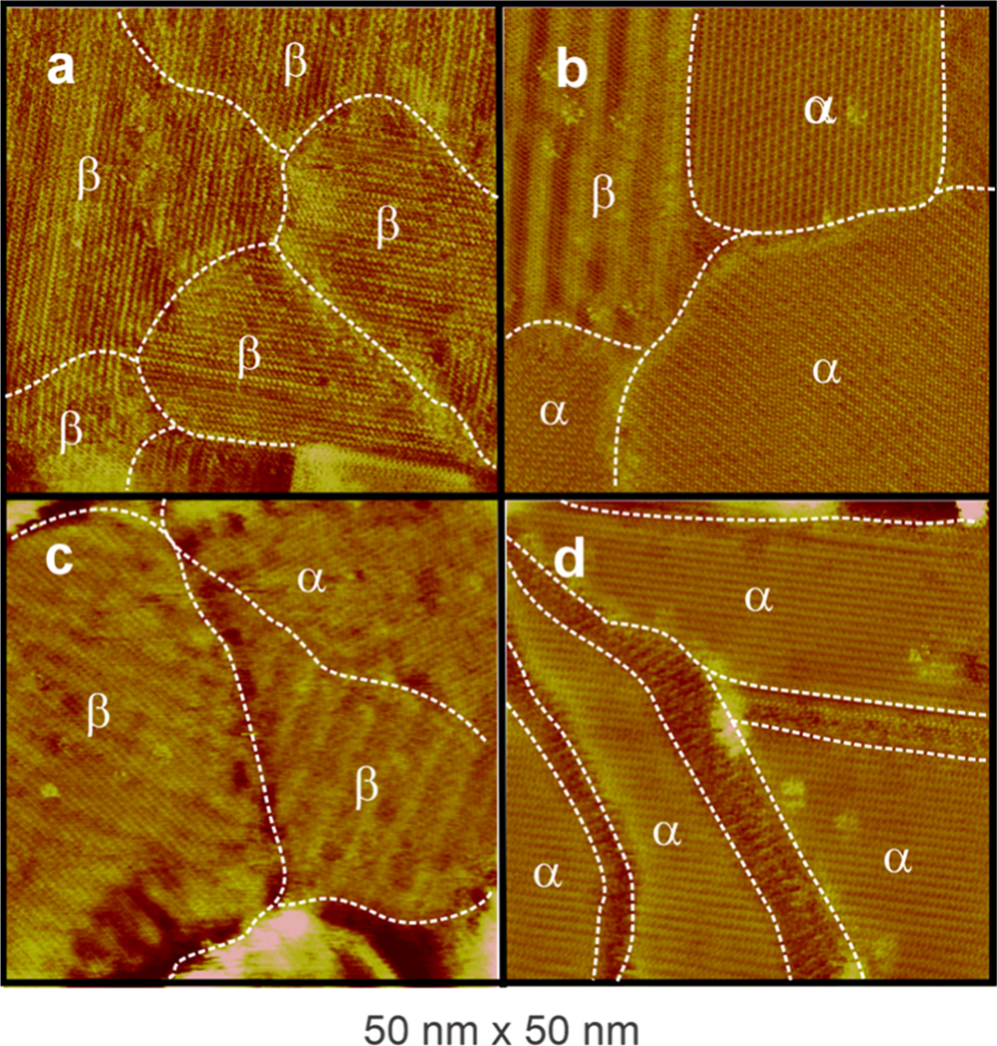
STM images showing the formation of two phases, α and β, on the BP*n* monolayers on the Au(100)–(1 × 1) surface. Terraces with smooth and striped domains are shown. (a) BP1 showing the β phase. (b) BP2 and (c) BP3 showing a mixture of α and β phases. (d) BP4 showing only the α phase. Note the fuzzy domain boundaries on BP4. STM parameters: bias voltage 200 mV, tunneling current 0.1 nA.

**Figure 3:**
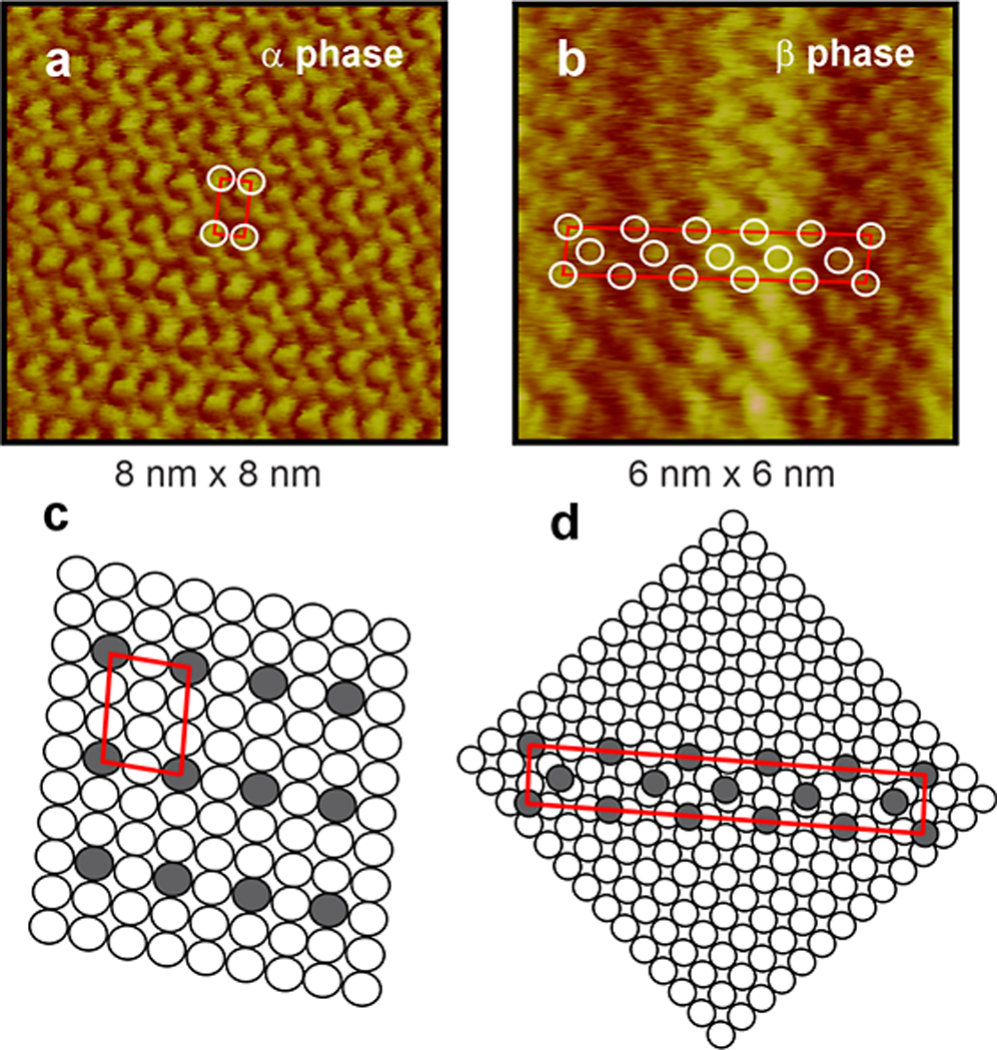
High-resolution STM images of BP*n* adsorbed on the Au(100)–(1 × 1) surface, showing details of the nearly (a) quadratic α phase and (b) β phase. Models according to the cell parameters for the (c) α phase and (d) β phase. STM parameters: bias voltage 50 mV, tunneling current 0.5 nA.

No vacancy islands are formed on the BP1 to BP4 monolayers on Au(100)–(1 × 1), in contrast to what is typically observed for alkanethiols^43,44^ or biphenyl-thiols^30–32,34,35,45^ on Au(111). However, for BP5 and BP6, vacancy islands are clearly nucleated on the gold terraces (Figure 4). The formation of vacancies for BP5 and BP6 suggests a deficiency of gold atoms during the adsorption process, particularly in the presence of long alkane chain BP*n* molecules. This points to a possible rearrangement of gold adatom–thiolate complexes^46^ rather than simple thiol adsorption, likely driven by strong lateral intermolecular interactions, which are much relevant for the development of the structural properties of the monolayers. The formation of gold vacancies is a key indicator of thiolate–Au_ad_–thiolate complex formation.^47^ In the case of BP5 and BP6, if such complexes are forming, gold adatoms must be extracted from terraces and/or step edges—a process that is known to be energetically favorable on the Au(100) surface.^48^ This would lead to a higher density of vacancies. Evidence of structural disorder is already noticeable in BP4 SAM formation, particularly at step edges and domain boundaries, as seen in the fuzzy domain edges in Figure 2d. The broad electrodesorption peaks observed for BP5 and BP6, along with the presence of a third voltammetric signal (see below), confirm that these SAMs exhibit more pronounced structural disordered and the formation of another surface chemical entity. Vacancy formation occurs because the development of the thiolate–Au_ad_–thiolate configuration requires a significant number of gold adatoms that are extracted from the terraces. This suggests that the formation of thiolate–Au_ad_–thiolate complexes is driven by strong lateral interactions, in particular for molecules with longer alkane chains. As a result, restricted molecular mobility leads to a disordered adlayer, as noted for BP5 and BP6 SAMs, where molecular resolution could not be achieved due to the absence of long-range ordered regions. However, on Au(111), even the formation of RS–Au_ad_–SR staples does not prevent the formation of well-ordered structures, indicating that additional factors contribute to the disordered adlayers of BP5 and BP6 on Au(100). One likely explanation is the adsorption energy, which is more favorable on the Au(100) facet,^49^ influencing the overall structural arrangement of the monolayer.

**Figure 4:**
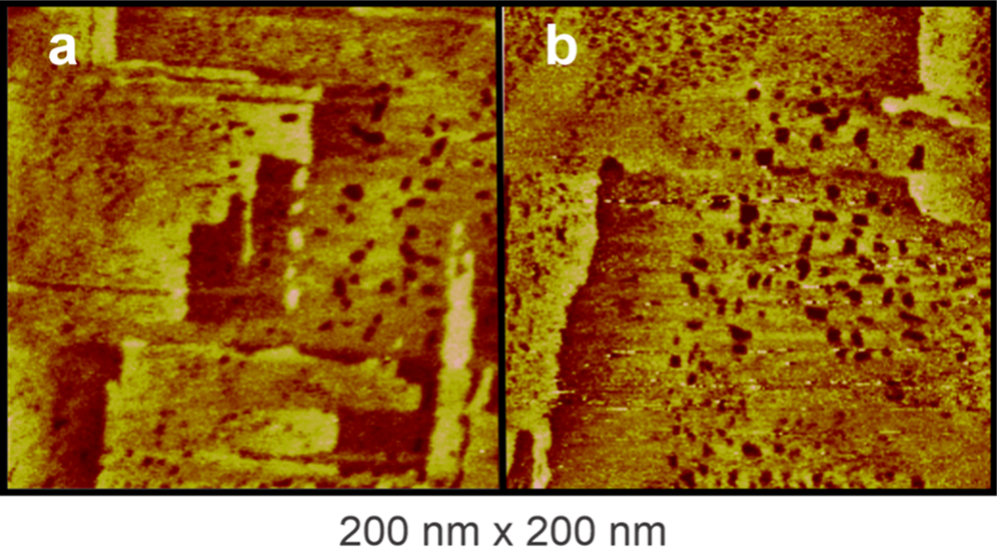
Large STM images showing high density of vacancy islands within domains of (a) BP5 and (b) BP6 adlayers on Au(100)–(1 × 1). Large terraces of gold islands are observed due to the lifting of the surface reconstruction. STM parameters: bias voltage 200 mV, tunneling current 0.1 nA.

### Charge Storage at the BP*n*-SAM/Au Electrode Interface

The ion permeability of the BP*n* monolayer was studied by using cyclic voltammetry (Figure SI1a) and immersion experiments (Figure 5). The adsorption properties of self-assembled monolayers can be precisely described by the capacitance of the double layer. SAMs form a dielectric barrier between the metal substrate and the electrolyte. The BP*n*-covered electrode exhibits nearly constant behavior over a wide potential interval (−0.6 V ≤ *E* ≤ 0.6 V), independent of the electrolyte used. This behavior is characteristic of thin organic adlayers with effective ion-blocking properties and minimal solvent penetration. A quantitative analysis of the capacitance as a function of the alkane spacer is depicted in Figure SI1b and the values are reported in Table 1. Within the uncertainty of the measurements, these results are consistent with values reported in the literature for alkanethiols^50,51^ and BP*n*-thiols on Au(111)–(1 × 1).^30,33^

**Figure 5:**
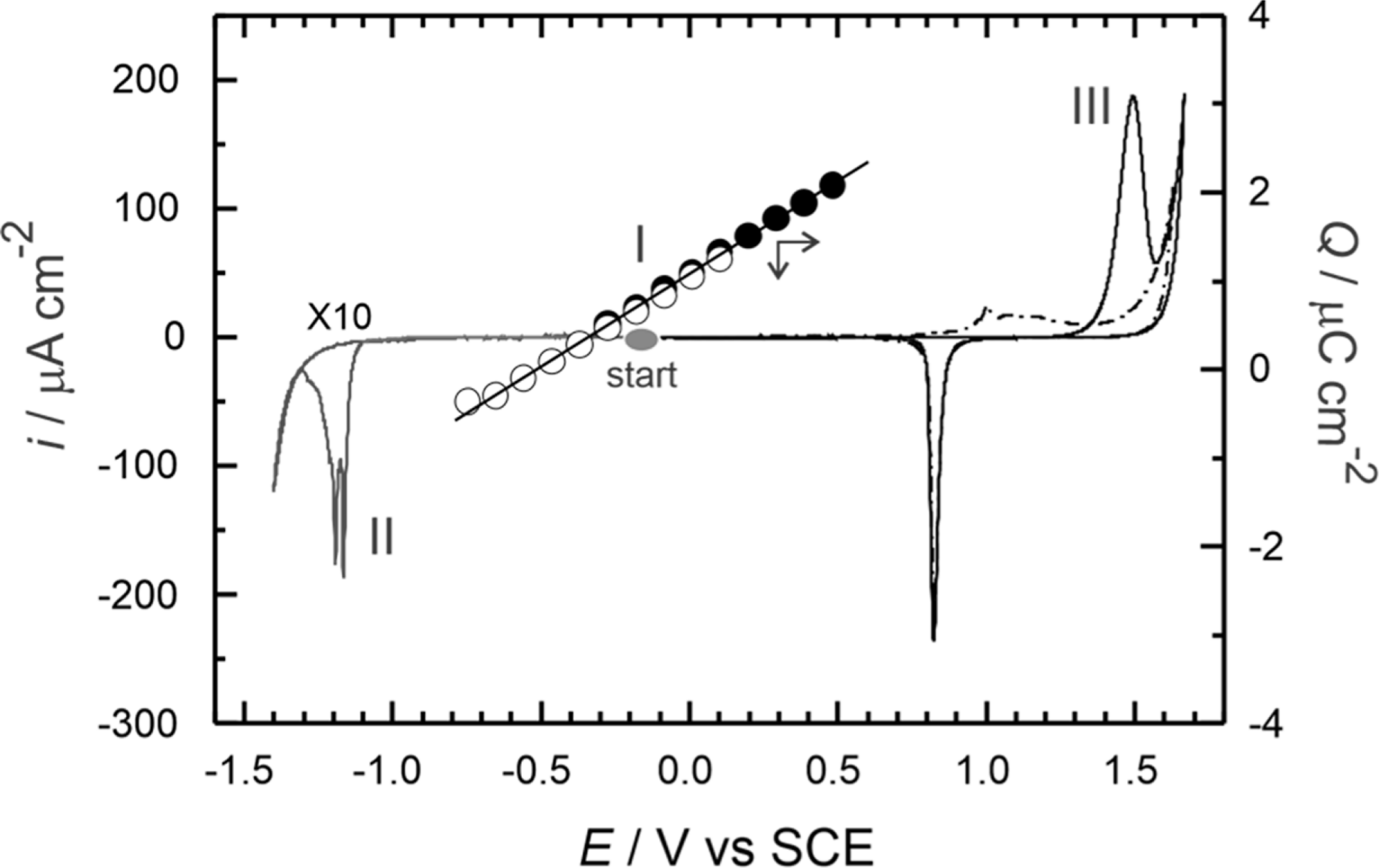
Complete picture of the electrochemical stability of the BP4 monolayer on Au(100)–(1 × 1) as a representative example of the BP*n* series. Region I illustrates the charges obtained from immersion experiments in 0.1 M NaOH (empty circles) and 0.1 M HClO_4_ (solid circles) at various potentials in the double-layer region. The black solid line represents the corresponding linear regression analysis of these data. Region II shows a typical current versus potential curve for the reductive desorption in 0.1 M NaOH (gray curve) which has been multiplied by ten for better visualization. The oxidative desorption zone in 0.1 M HClO_4_ is shown in region III. The dashed trace represents the second cycle of the oxidative desorption. The starting potential for both desorption processes is set within the double-layer region and is represented by the gray circle. Scan rate: 10 mV s^–1^.

**Table 1:** Characteristic Electrochemical Data for BP*n* Monolayers on Au(100)–(1 × 1) in the Double-Layer Region, for Reductive (in 0.1 M NaOH) and Oxidative (in 0.1 M HClO_4_) Desorption

molecule	*C*^a^μF cm^–2^	*C*^b^μF cm^–2^	*E*_PZC_ V (SCE)	*E*_P1_ V (SCE)	*E*_P2_ V (SCE)	*Q*_red_ μC cm^–2^	θ
BP1	3.1 ± 0.2	2.8 ± 0.2	–0.31 ± 0.03	–1.157	–1.261	88 ± 5	0.46 ± 0.02
BP2	3.0 ± 0.1	2.6 ± 0.2	–0.42 ± 0.03	–1.183	–1.280	100 ± 7	0.52 ± 0.03
BP3	2.6 ± 0.1	2.4 ± 0.1	–0.45 ± 0.02	–1.155	–1.278	90 ± 6	0.46 ± 0.03
BP4	2.2 ± 0.2	2.1 ± 0.1	–0.47 ± 0.01	–1.166	–1.195	97 ± 6	0.50 ± 0.03
BP5	1.9 ± 0.2	1.9 ± 0.2	–0.47 ± 0.02	–1.179	–1.257	85 ± 5	0.44 ± 0.02
BP6	2.0 ± 0.2	1.8 ± 0.2	–0.46 ± 0.03	–1.181	–1.271	100 ± 8	0.52 ± 0.04

a Calculated using the immersion technique.

b Calculated from cyclic voltammetry measurements.

To further investigate the quality of the BP*n* adlayers, we measured the interfacial capacitance by the so-called “immersion technique”.^30,52^ In this method, the electrode potential was strictly controlled as the dry BP*n*-covered surface was carefully approached to the electrolyte in an Ar atmosphere, until a hanging-meniscus configuration is formed, avoiding wetting the upper-walls of the electrode. The charging current initially increases or decreases steeply and then exponentially decays to zero. The charge consumed during this process can be attributed to the formation of an electrochemical double layer at the respective immersion potentials. Plots of charge density versus potential, obtained by integrating the current transients, show a linear relationship with a slope that excellently correlates with the capacitance values obtained from linear potential scans. This confirms that BP*n* thiols form compact monolayers on Au(100)–(1 × 1). As an example, Figure 5 shows open and filled circles symbolizing the charges calculated using the immersion technique in 0.1 M NaOH or 0.1 M HClO_4_, respectively. Moreover, thiol adsorption on metal surfaces leads to a shift in the potential of zero charge (*E*_PZC_) compared with the clean metal surface. An estimation of the *E*_PZC_ of the BP4-covered Au(100)–(1 × 1) electrode is identified by the zero-crossing of this plot. The estimated value is significantly more negative (−0.45 ± 0.01 V) than the *E*_PZC_ of the bare Au(100)–(1 × 1) surface (+0.08 V).^23^

### Electrochemical Stability

The reductive potentials represent thermodynamic free energies of desorption and, thus, are directly related to the electrochemical stability of the monolayers. For BP*n*, this stability is limited at both negative and positive potentials by reductive and oxidative desorption, respectively. Electrodesorption measurements were performed by using cyclic voltammetry, which also provides a quantitative assessment of the thiol surface concentration. Figure 6 presents the cyclic voltammograms of the reductive desorption of a series of BP*n*-thiol (*n* = 1–6) monolayers from Au(100)–(1 × 1) in 0.1 M NaOH, illustrating the electrochemical stability of these monolayers. Before measurement, the BP*n*-coated electrode was carefully immersed into the electrolyte solution under strict potential control at a voltage of −0.2 V under a hanging meniscus configuration. The potential was then scanned from −0.2 to −1.45 V to reduce the chemisorbed thiols. For BP1 to BP4, two reductive current peaks were observed without an anodic counterpart at a scan rate of 10 mV s^–1^. In contrast, for BP5 and BP6 monolayers, three sharp features were observed. When the potential was scanned back to positive values, no counter anodic peaks were detected. Previous studies on BP*n* monolayers formed on Au(111)^30,32,33^ reported a single, narrow cathodic wave for BP1 to BP4 adlayers, also without an anodic counterpart. Likewise, in those studies, the BP5 and BP6 adlayers presented different features, including notable oxidative readsorption upon scanning the potential back to positive values. The presence of multiple cathodic waves can be attributed to the immediate products formed after the reductive desorption of alkanethiols as well as the influence of surface crystallography.

**Figure 6:**
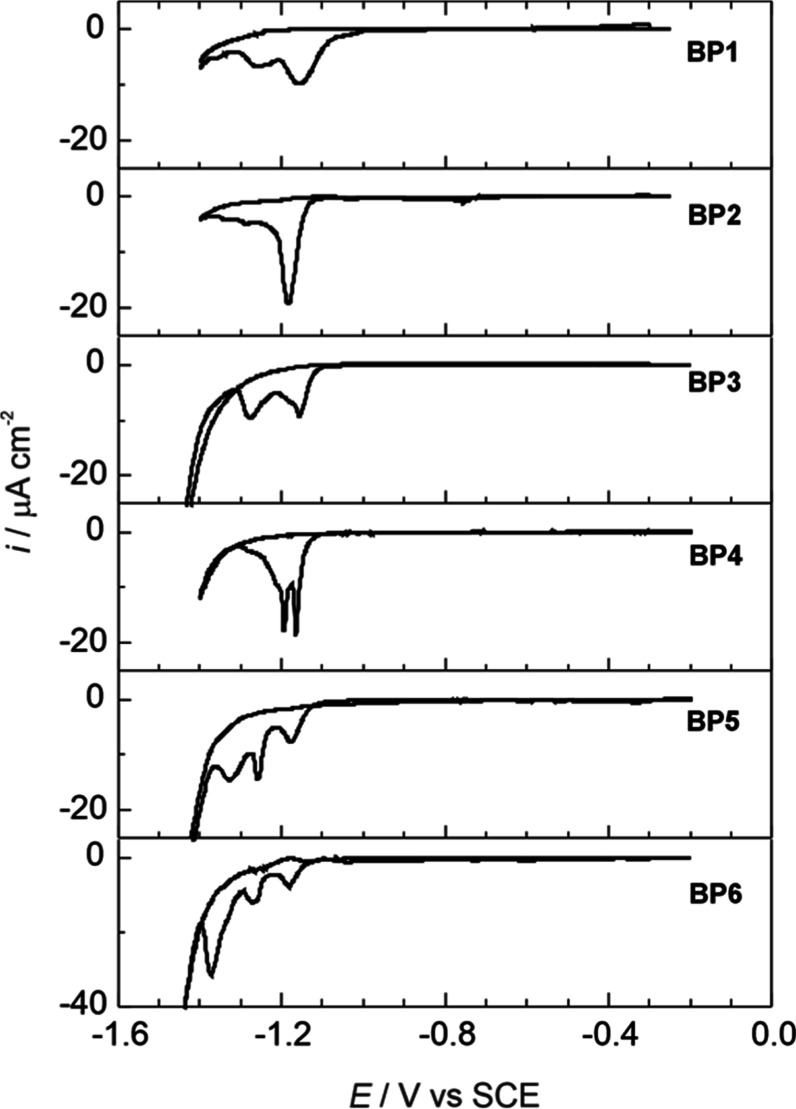
Current–potential curves for the reductive desorption of biphenyl-thiol (BP*n*, *n* = 1–6) monolayers on Au(100)–(1 × 1) in 0.1 M NaOH after immersion at −0.2 V. Scan rate: 10 mV s^–1^.

Previously, it has been established^53^ that desorption from stepped surfaces introduces additional desorption peaks at more negative potentials compared to smoother surfaces. Moreover, the multiple electrodesorption waves observed nicely correlate with the distinct structural properties of the Au(100)–(1 × 1) surface, as revealed by STM analysis.

A higher electrochemical stability has been reported for aliphatic and aromatic thiols desorbed from Au(100) compared to those detached from Au(111).^20^ Similarly, for BP*n* molecules, we found greater electrochemical stability on Au(100)–(1 × 1), with the peak potential appearing beyond −1.1 V for all monolayers (Figure 7a). The reductive potential values of BP*n* are more negative that those reported for alkanethiol adlayers on Au(100)^24^ and for BP*n* on Au(111).^30^ This difference between crystallographic planes could be explained in terms of the more negative work function (or potential of zero charge, PZC) of Au(100) compared to Au(111). According to Ref 23, the PZC value of bare Au(100)–(1 × 1) (0.08 V vs SCE) is shifted by approximately −0.15 V relative to that of the bare Au(111)–(1 × 1) (0.23 V vs SCE) surface in the supporting electrolyte. This shift correlates well with the more negative PZC values obtained for BP4 on Au(100) (−0.47 V) with respect to Au(111) (−0.3 V).^30^ These findings are consistent with density functional theory (DFT) studies^54^ by Masens et al., which indicate increased thermodynamic stability of the Au(100)–S bond for thiol adsorption compared to Au(111).

**Figure 7:**
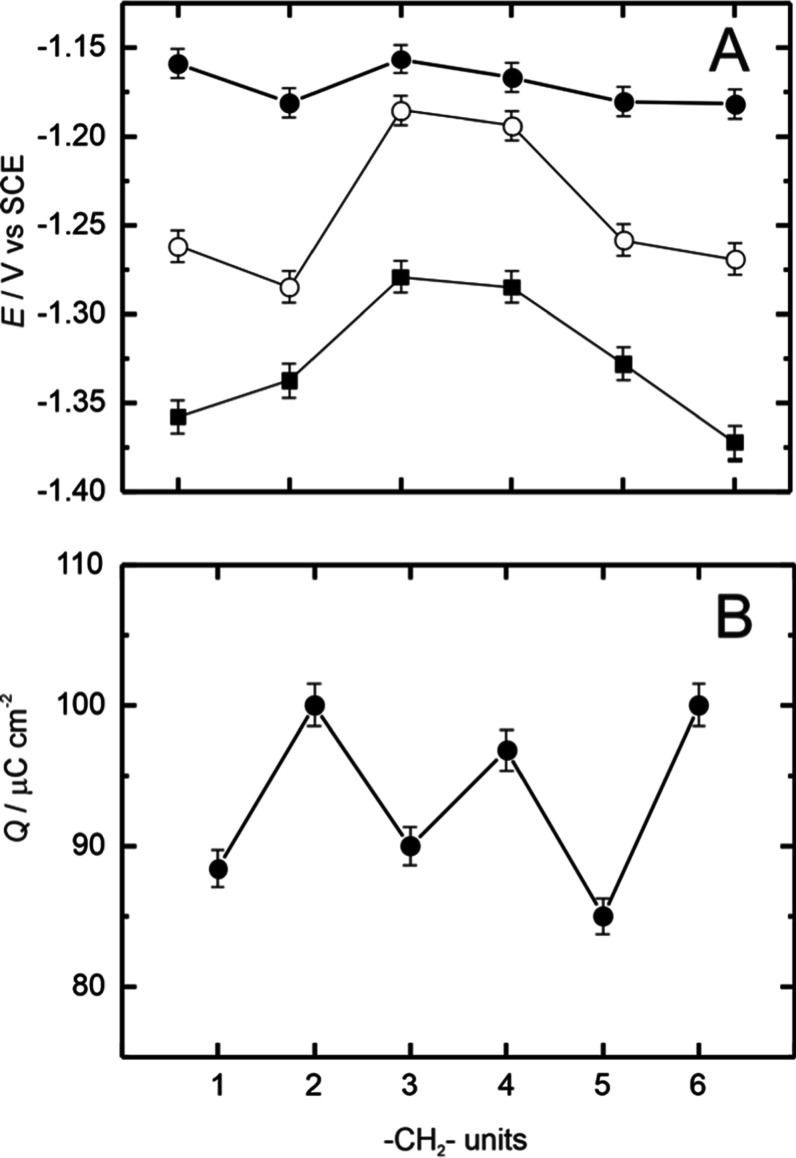
Chain length dependence of the potentials and charges obtained for the reductive desorption of Biphenyl-thiol (BP*n*, *n* = 1–6) monolayers on Au(100)–(1 × 1) in 0.1 M NaOH (values in Table 1).

A further comparison of the features observed in the present work highlights that the potential of the first reductive desorption peak of BP1 to BP4 exhibits an odd–even effect similar to that reported for the BP*n*/Au­(111) electrode (Figure 7a). However, BP5 and BP6 deviate from this sequence, which could be related to increased defect density, monolayer disorder (as indicated by the STM results), and variations in surface chemistry during reductive desorption. This suggests that different molecular interactions occur with longer alkane chains in BP*n*. In fact, both experimental and theoretical evidence^46^ derived from in situ XPS and DFT studies have recently demonstrated that the mechanism immediately following the detachment of an alkanethiol molecule from the surface may differ depending on the crystallographic orientation. On Au(111), this process tends to involve the formation of dialkyl sulfide (RS−), whereas in Au(100), it primarily leads to the formation of dialkyl disulfide (RS–SR) and thiol, following the generation of an alkyl sulfide radical (RS^•^). At the initial stages of the desorption process, a gold adatom remains attached to the thiol on the Au(111) and Au(100) surfaces. On Au(111), the S–Au bond breaks when sulfur is positioned 2.1–2.2 Å away from the metal surface, with an energetic stabilization occurring as the gold adatom reapproaches the surface. In the case of Au(100), the gold adatom begins to return to the surface when sulfur has been pulled by only 1.6 Å, and no clear energetic stabilization is observed. Under this scenario, the distance between the alkyl sulfide radical (RS^•^) and the nearest sulfur atom is 4.3 Å on Au(111) but only 2.3 Å on Au(100). At this shorter distance, thiol molecules tend to occupy top or near bridge positions, where a local minimum in energy favors their adsorption on these positions.^55^ Given that the typical S–S bond length is 2.05 Å, this proximity increases the probability of RS–SR bond formation near the Au(100) surface, whereas such bonding is not as favorable on Au(111).^46^ This phenomenon could also explain why BP5 and BP6 exhibit three desorption peaks. Once detached from the Au(100), these molecules, being more hydrophobic, diffuse more slowly into the bulk electrolyte. This prolonged residence time enhances their ability to form chemical species through strong lateral interactions between reduced and unreduced thiols, a process further triggered by increased ionic permeability. Nevertheless, the total charge distribution below the current peaks certainly exhibits a pronounced odd–even alternation as the aliphatic chain length increases across the BP*n* series (Figure 7b). The reductive charge was calculated by integrating the voltammetric waves after background correction. For the odd-numbered series, the charge ranges between 87 ± 5 μC cm^–2^, while for the even-numbered series, this value is 98 ± 5 μC cm^–2^. Within the experimental uncertainties, these values nicely correlate with previously reported values for hexanethiol on Au(100)–(1 × 1),^24^ other *n*-alkanethiols on Au(111),^50,56^ and those reported for the same BP*n* on Au(111).^30^ Comparing the two series, the higher charge values for the even-numbered BP*n* molecules correlate well with the predominance of the more compact α-phase present in these monolayers. The increased charge values may also explain the enhanced electrochemical stability of the BP*n*-thiols on Au(100)–(1 × 1), as they suggest a higher surface concentration of adsorbed molecules.

Assuming that the reduction of one thiol molecule requires one electron,^57^ according to RS – Au + 1e^–^ ⇄ RS^–^ + Au, the thiol surface concentration is 9 ± 0.2 × 10^–10^ mol cm^–2^ for the odd-numbered series (θ = 0.46, 0.46, 0.44) and 10 ± 0.4 × 10^–9^ mol cm^–2^ for the even-numbered series (θ = 0.52, 0.50, 0.52). The higher surface concentration for the even-numbered series indicates a better blocking ability and explains its slightly greater electrochemical stability. These coverage values agrees well with those reported for hexanethiolate SAMs on Au(100)–(1 × 1).^25^ This difference in coverage between BP-odd and BP-even can be attributed to the higher packing density of the α-phase observed in BP*n*-even monolayers, compared to the less densely packed β-phase in BP*n*-odd monolayers (Figure 3), at least for BP1 to BP4. Additionally, the broader desorption peak observed for BP*n* odd suggests a higher degree of disorder in these SAMs compared to BP*n*-even.

### Barrier Properties and Charge Transfer Studies

The charge transfer properties of the BP*n* adlayers are kinetically limited and governed by electron tunneling. The efficient blocking properties of the monolayers hinder ion transfer on these surfaces, and it strongly depends on the length of the aliphatic spacer, thereby reducing the rate of electron transfer across the monolayer. These measurements were performed in the presence of a redox probe, in 1 mmol L^–1^ K_3_Fe­(CN)_6_/K_2_Fe­(CN)_6_ in 0.1 M KClO_4_ using freshly BP*n*-covered and bare Au(100) electrodes (see Supporting Information). To evaluate the decay constant (β) for electron tunneling, the logarithm of the tunneling current was plotted against the molecular length (inset, Figure SI2b). The estimated β value was (1.0 ± 0.1) per −CH_2_– unit over a potential range between −0.5 and 0.4 V. Given a thickness change of 0.13 nm per −CH_2_– group,^58^ this corresponds to β = (7.7 ± 0.02) nm^–1^, consistent with reported values for electron transfer across hydrocarbon spacers in donor–acceptor systems.^59^ Assuming a simple rectangular tunneling barrier and a monolayer with minimal defect contributions, the effective barrier height is approximately (0.87 ± 0.03) eV,^59^ which is lower than that reported for the Au(111)–(1 × 1) surface. This suggests that the nature of the S–Au bond governs charge transport through the monolayer as is largely independent of the alkane chain length.

## Conclusions

The present study demonstrates that the structural, molecular packing density, and electrochemical properties of BP*n* monolayers are significantly influenced by the crystallography of the substrate. STM analysis revealed the existence of two distinct molecular phases (α and β) for shorter alkane chains (BP1–BP4), whereas longer alkane chains (BP5, BP6) exhibited a disorder structure. This implies complex surface chemistry, representing a missing piece in the understanding of these systems. As the alkane chain length increases, van der Waals interactions become more dominant, ultimately overriding the influence of the S–Au in determining the molecular structure of the adlayer on Au(100)–(1 × 1). The potential of zero-charge values estimated for the BP*n*–Au­(100)–(1 × 1) surface is significantly more negative (−0.45 V) compared to the bare Au(100)–(1 × 1) electrode (+0.08 V). This finding highlights the possibility of tuning the electronic properties of metal surfaces through the simple formation of SAMs. Moreover, BP*n* thiols adsorbed on Au(100)–(1 × 1) exhibit higher electrochemical stability than the same molecules previously reported on Au(111)–(1 × 1), as indicated by the more negative desorption potentials. For short-chain alkanethiols (BP1 to BP4), the first reduction peak follows an odd–even effect in the stability of reductive desorption, whereas BP5 and BP6 deviate from this pattern. The charge densities associated with reductive desorption exhibit a pronounced odd–even effect. The BP*n* adlayers with an even number of methylene units are more stable than those with an odd number, reflecting the higher packing density and the formation of long-range ordered SAMs. These findings underscore the crucial role of substrate crystallography and reveal how the molecule–substrate interface governs both the structural and the functional properties at the interface.

## Supplementary Material



## Notes

Capacitance measurements and charge transfer properties of BP*n* (*n* = 1–6) SAMs on Au(100)–(1 × 1) (PDF)

## ∥

College of Chemistry and Chemical Engineering, Hunan University, Changsha 410082, China
