# Supplementary material for: Structural and Electrochemical Properties of 4‑Methyl-4′‑(n‑mercaptoalkyl) Biphenyls Self-Assembled on the Au(100)–​(1 × 1) Surface
Source: Langmuir. 2025 Jul 29;41(31):20648–56. doi: 10.1021/acs.langmuir.5c02088 (PMC12356081; doi:10.1021/acs.langmuir.5c02088)
Supplement: Supplementary file 1 [file la5c02088_si_001.pdf]

Supporting Information

Structural and electrochemical properties of 4–methyl–4′–(n–mercaptoalkyl) biphenyls self-assembled on the Au(100)–(1×1) surface

R. Aguilar-Sánchez[1,2*], Yongchun Fu[3†], M. Homberger[2], U. Simon[2]

[1] *Facultad de Ciencias Químicas, Benemérita Universidad Autónoma de Puebla. 72570 Puebla, México.*
[2] *Institute of Inorganic Chemistry, RWTH Aachen University. 52074 Aachen, Germany.*
[3] *Department of Chemistry and Biochemistry, University of Berne, CH-3012 Berne, Switzerland.*

The reciprocal of the capacitance (C^{-1}) shows a linear increase relationship with the spacer chain length of the molecule, and thus lower capacity. The solid line represents the linear regression analysis of C^{-1}, which was calculated at –0.4 V and a scan rate of 50 mV s^{-1} in 0.1 M NaOH. The slope of this line is 0.042±0.002 μF^{-1} cm^2 per CH_2 group, with an intercept of 0.297±0.009 μF^{-1} cm^2. The linearity found, suggests a high degree of organization within the adlayer and a poor solvent accessibility. The recorded capacitance values imply that the ion permeability of the BPn layer is not significantly affected by the substrate crystallography.

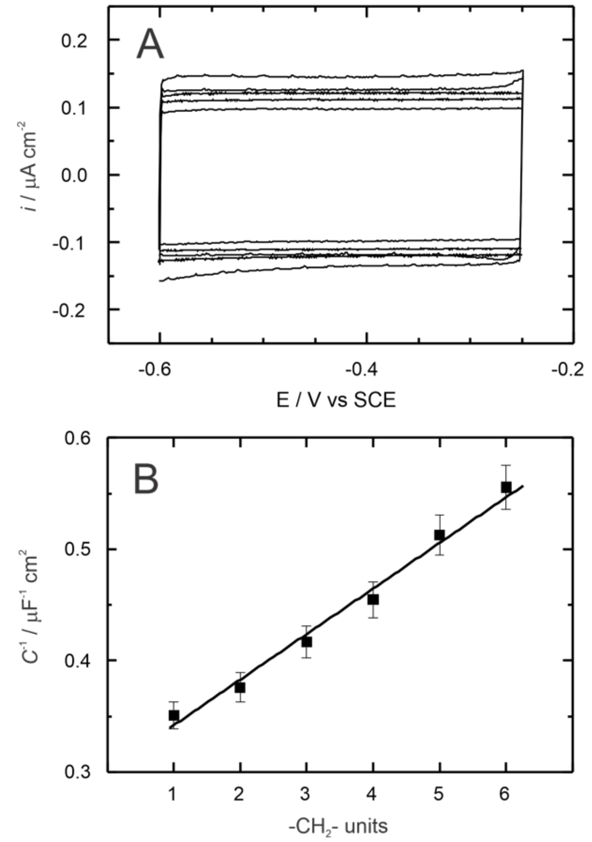

Fig. SI-1 (A) Double layer capacitance of BPn (n = 1 – 6) SAMs on Au(100)–(1×1) in 0.1M NaOH calculated from cyclic voltammograms at a scan rate of 50 mV s^{-1} in the double layer region. (B) Linear dependence of reciprocal capacitance with respect to molecular alkanechain length of BPn.

To study the effect of the adsorbed layer on the charge transfer properties of the BPn adlayers, we conducted experiments in the presence of a redox probe, 1 mmol L^{-1} $K_3Fe(CN)_6/K_2Fe(CN)_6$ in 0.1 M KClO4. Each measurement started at the open circuit potential of the redox-active species (0.15 V vs SCE), using freshly prepared monolayers. Figure SI-2a shows a series of voltammograms for the oxidation and reduction of $Fe(CN)_6^{4-/3-}$ in the presence of BPn adlayers with varying lengths. For comparison, the voltammogram of the bare Au(100) electrode is also included (dashed line). As observed in Figure SI-2a, there is a dramatic difference in the voltammetric response of the BPn-covered and bare Au(100) electrode. Whereas the bare Au(100) electrode exhibits a peaked shape voltammogram characteristic of a diffusion-controlled process, the presence of BPn SAMs leads to a notable decrease in current and a splitting of the peak potential, which becomes more pronounced with increasing chain length of the aliphatic spacer. This behavior is consistent with a process which is kinetically limited governed by electron tunneling and strongly depends on the length of the aliphatic spacer, thereby reducing the rate of electron transfer across the monolayer. The efficient blocking properties of the monolayers hinder ion transfer on these surfaces. If electron tunneling is implicated in the electron transfer, the current should decrease exponentially with the length of the aliphatic moiety according to $\beta = (\partial \ln i/\partial (CH)_2)_{E=\text{ const}}$. From this analysis, the decay constant β for electron tunneling was evaluated by plotting the logarithm of the tunneling current versus the molecular distance (inset Fig. SI-2b). The estimated β value was (1.0±0.1)/CH_2–unit and, given a thickness of 0.13 nm/–CH_2– group, β corresponds to (7.7±0.02) nm^{-1}, which is consistent with reported values for electron transfer in donor-acceptor pairs separated by hydrocarbon linkages. This parameter significantly depends on the molecular structure of the adlayer and can serve as an indicator to evaluate its electron tunneling efficiency.

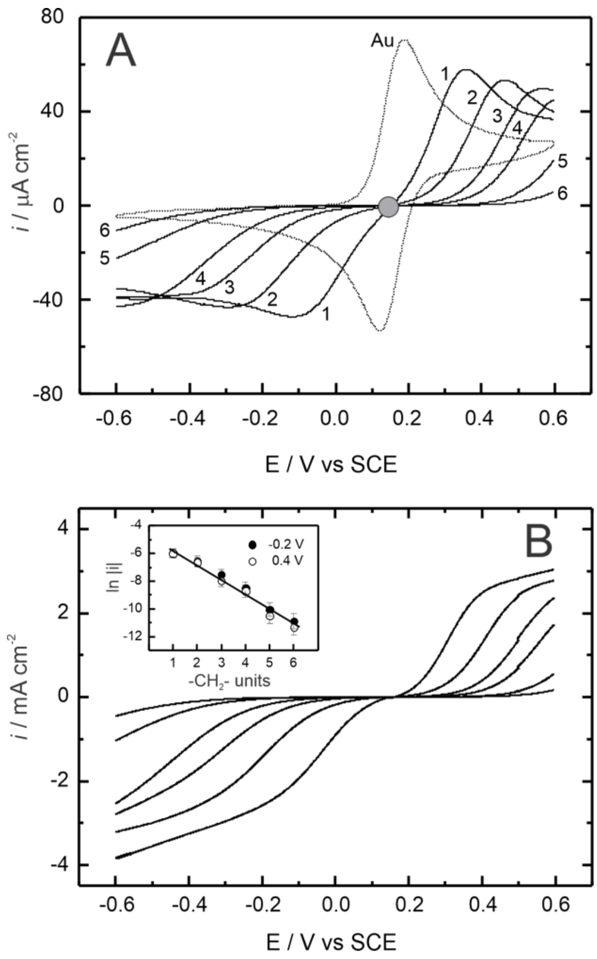

Fig. SI-2 (A) Current-potential curves for the oxidation-reduction of 1mM $[Fe(CN)_6]^{4-/3-}$ in 0.1 M $KClO_4$ on freshly prepared BPn monolayers on Au(100)–(1×1). For comparison, the gray curve represents the cyclic voltammogram for the bare electrode Au(100)–(1×1) in the same solution. (B) The effect of the reactant depletion due to diffusion was corrected by a convolution analysis for all BPn monolayers. The inset shows the linear dependence of the kinetic current versus the alkanechain length.
